# Genome-wide assessment of population structure and genetic diversity and development of a core germplasm set for sweet potato based on specific length amplified fragment (SLAF) sequencing

**DOI:** 10.1371/journal.pone.0172066

**Published:** 2017-02-10

**Authors:** Wenjin Su, Lianjun Wang, Jian Lei, Shasha Chai, Yi Liu, Yuanyuan Yang, Xinsun Yang, Chunhai Jiao

**Affiliations:** 1 Food Crops Institute, Hubei Academy of Agricultural Sciences, Wuhan, China; 2 Beijing Key Laboratory of Crop Genetic Improvement/Laboratory of Crop Heterosis and Utilization, Ministry of Education, China Agricultural University, Beijing, China; 3 College of Agriculture, Yangtze University, Jingzhou, China; 4 Hubei Academy of Agricultural Sciences, Wuhan, China; Saint Mary's University, CANADA

## Abstract

Sweet potato, *Ipomoea batatas* (L.) Lam., is an important food crop that is cultivated worldwide. However, no genome-wide assessment of the genetic diversity of sweet potato has been reported to date. In the present study, the population structure and genetic diversity of 197 sweet potato accessions most of which were from China were assessed using 62,363 SNPs. A model-based structure analysis divided the accessions into three groups: group 1, group 2 and group 3. The genetic relationships among the accessions were evaluated using a phylogenetic tree, which clustered all the accessions into three major groups. A principal component analysis (PCA) showed that the accessions were distributed according to their population structure. The mean genetic distance among accessions ranged from 0.290 for group 1 to 0.311 for group 3, and the mean polymorphic information content (PIC) ranged from 0.232 for group 1 to 0.251 for group 3. The mean minor allele frequency (MAF) ranged from 0.207 for group 1 to 0.222 for group 3. Analysis of molecular variance (AMOVA) showed that the maximum diversity was within accessions (89.569%). Using CoreHunter software, a core set of 39 accessions was obtained, which accounted for approximately 19.8% of the total collection. The core germplasm set of sweet potato developed will be a valuable resource for future sweet potato improvement strategies.

## Introduction

Sweet potato (*Ipomoea batatas* (L.) Lam.) is a crop of considerable economic and social importance in developing countries [1]. Because of its high productivity and abundant protein, calorie and vitamin contents, it plays a key role in alleviating hunger and malnutrition in impoverished areas [2]. In China, sweet potato has been ranked fourth in importance among staple crops; in 2010, it was grown on approximately 3.65 million ha, with a total annual yield of 21.26 t.ha^-1^, occupying approximately 45.1% of the worldwide sweet potato planted area and accounting for approximately 75.3% of the worldwide total annual sweet potato production [3].

Genetic resources are of paramount importance for crop improvement. Studies from archaeology, linguistics, history and biotechnology indicate that Central and South America is the primary center of sweet potato diversity, with East Africa, Asia and Oceania suggested as the secondary centers of diversity [4,5]. Over time, natural hybridization and selection have resulted in the evolution of different kinds of native sweet potato cultivars and a magnitude gene pool is reserved in China [6]. In the 1970s, China made significant progress in sweet potato breeding, which led to the release of many cultivars, including the excellent cultivar Xushu 18, which is the offspring of the most important exotic cultivars: Okinawa 100 from Japan and Nancy Hall from the United States [7]. However, the recurrent use of a few elite lines as parental stocks has decreased genetic diversity and narrowed the genetic background for sweet potato improvement [8]. Additionally, a large number of germplasm resources have been conserved, but their use is limited due to an unmanageable number of accessions. As a result, genetic diversity analyses and core germplasm development have been proposed to better manage these collections [9].

Genomic tools, such as molecular markers, can help elucidate the genetic background of the accessions, which could support breeding strategies. Random amplified polymorphic DNA (RAPD) [10,11], amplified fragment length polymorphisms (AFLPs) [12,13] and inter-simple sequence repeats (ISSRs) [14,15] are frequently used to fingerprint and characterize sweet potato varieties. However, these types of markers are far from saturated due to an insufficient number of markers. Simple sequence repeats (SSRs) are co-dominant markers that are more saturated [16]. A previous study of SSRs in 380 accessions provided a foundation upon which to study the genetic diversity of the germplasm at a fine scale [17]. SNPs are more useful than conventional markers because they are the most abundant and stable type of genetic marker in most genomes [18]. In recent years, deep sequencing technology has been rapidly developed to exploit these advantages and has enabled the high-throughput identification of SNPs [19–23], albeit with the disadvantage of becoming cost-prohibitive when the population is large. A new strategy for *de novo* SNP discovery and genotyping of large populations, referred to as specific length amplified fragment (SLAF) sequencing (SLAF-seq) [24], was recently reported. This high-resolution method has been tested on many organisms, including soybean, sesame, cucumber and the common carp (*Cyprinus carpio* L.), whose genome sequence was not reported at the time of publication [24–27].

This study presents a comprehensive view of the genome-wide variation among 197 sweet potato accessions, most of which are from China, and provides a core germplasm set representing the maximum diversity of the total collection. The described core set can be more efficiently used for breeding than the whole collection.

## Materials and methods

### Plant varieties and DNA extraction

A set of 197 sweet potato accessions was evaluated in the present study. These accessions were generated from different agro-climatic zones and were cultivated on the Experimental Farm of Hubei Academy of Agricultural Sciences in Jiangxia District, Wuhan, China, in 2015. The experimental site (29°18’N latitude and 113°42’E longitude with an altitude of 20–40 meters above the sea level) had a humid climate.

**Fig 1:**
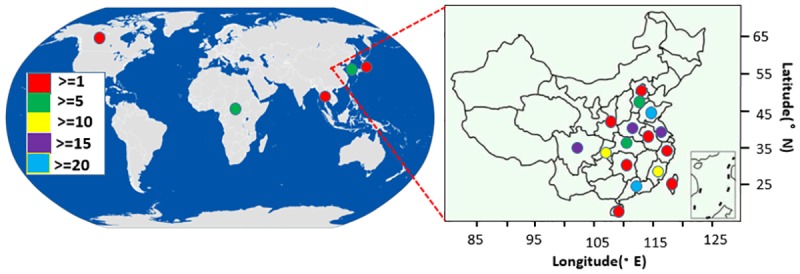
Location of the sweet potato accessions from around the world, highlighting China.

The samples included 50 landraces and 147 modern cultivars. Among these accessions, 178 came from China, 6 from Africa, 2 from Japan, 8 from South Korea, 1 from Thailand, and 2 from the USA (Fig 1). Detailed information on the regional distribution of the 197 accessions is provided in S1 Table.

Bulked young healthy leaves from each accession were collected, frozen in liquid nitrogen and used for DNA extraction. DNA was isolated via the CTAB protocol [28]. The DNA concentration was quantified using a NanoDrop-2000 spectrophotometer, and DNA samples were diluted to 50 ng μL^-1^.

### High-throughput sequencing and data processing

Genomic DNA was analyzed according to the SLAF-seq method. To obtain evenly distributed SLAF tags and to avoid repetitive SLAF tags for maximum SLAF-seq efficiency, simulated restriction enzyme digestion was carried out *in silico*. The genomic DNA of the materials was digested with the *Rsa*I restriction enzyme, and *Arabidopsis thaliana* DNA was used as a control to assess the normal rate of enzyme digestion [29]. The SLAF library was constructed according to procedures described by Sun with a few modifications [24]. DNA fragments of 264–314 bp were selected as SLAFs and prepared for paired-end sequencing on the Illumina High-Seq 2500 sequencing platform (Illumina, Inc.; San Diego, CA, US) at Beijing Biomarker Technologies Corporation.

The raw reads were clustered based on similarity above 90%. The SLAF tags were defined as the group with the most samples. The samples with the most tags were used as references, and G_ATK_ [30] and S_AMTOOLS_ [31] were employed for SNP calling. Only SNPs called by both G_ATK_ and S_AMTOOLS_ were considered to be of high quality.

### Statistical analyses

The raw data were qualified [29], and these data can be further used for data mining and additional analyses. A total of 62,363 SNPs from 197 accessions were developed to calculate genetic structure and relationships (S2 Table). ADMIXTURE was employed to investigate population structure based on the maximum-likelihood method; five independent simulations were carried out for each *K* (number of groups) ranging from 1 to 10, and accessions were assigned to a corresponding population based on their maximum membership probabilities [32]. A phylogenetic tree based on the neighbor-joining method and a UPGMA dendrogram based on Nei’s distance were constructed in MEGA 5 [33,34] using the developed SNPs. A principal component analysis (PCA) was performed with Cluster software [35,36].

Genetic diversity, polymorphic information content (PIC), and the minor allele frequency (MAF) were calculated using calculation scripts developed by Biomarker Technologies Corporation. The presence of molecular variance among groups, among accessions within groups and within accessions was assessed via analysis of molecular variance (AMOVA) using Arlequin [37]. Furthermore, pairwise levels of differentiation were estimated using the PopGen package in BioPerl. Finally, CoreHunter software was used to develop a core germplasm set [38,39].

## Results

### Population structure and phylogenetic relationships

**Fig 2:**
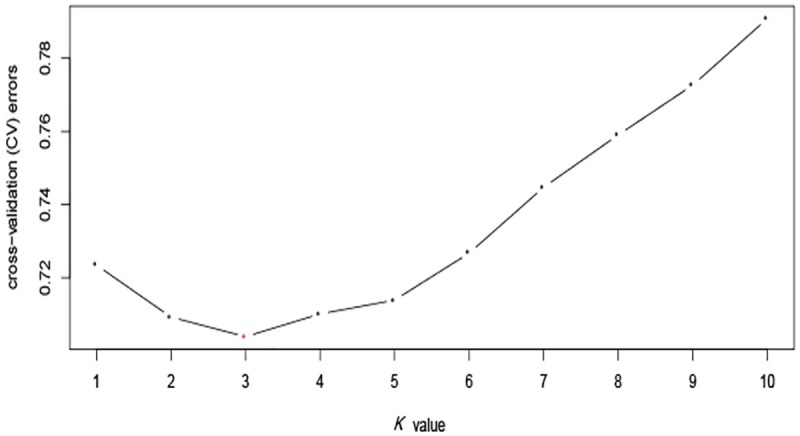
ADMIXTURE estimation of the number of groups for *K* values ranging from 1 to 10.

**Fig 3:**
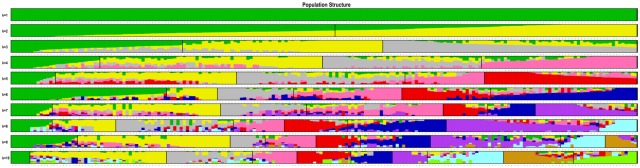
Pattern of variation among the 197 accessions based on 62,363 SNPs. The x-axis showed different accessions. The y-axis quantified the membership probability of accessions belonging to different groups. Colors in each row represented structural components.

The estimated membership fractions of the 197 accessions for different values of *K* ranged from 1 to 10, and the maximum likelihood revealed by the population structure showed an optimum value of 3 (*K* = 3) (Figs 2 and 3), which indicated that the entire population could be categorized into three groups: group 1, group 2 and group 3. Group 1 contained 54 accessions, one of which was from Africa, while the remainder were from different provinces in China. Group 2 contained 63 accessions from China. Group 3 contained 80 accessions, 18 of which were from Africa, Japan, South Korea, and Thailand, while the remainder were from different provinces in China (Table 1). Among the modern cultivars, 54 accessions were classified into group 1, 13 into group 2, and 80 into group 3. Among the landraces, 50 accessions were assigned to group 2 (Table 2). For a more thorough classification of the model-based structure relative to prior grouping information, the following analyses were based on the results of model-based population structure.

**Table 1:** Regional distribution of the total collection and the core set in this study.

Collection region	Group			Number of core sets
	Group 1	Group 2	Group 3	
Africa	1	0	5	1
Anhui	3	0	0	0
Beijing	1	0	2	0
Chongqing	7	0	4	2
Fujian	0	3	8	2
Guangdong	0	47	5	8
Hainan	0	3	0	2
Hebei	3	0	4	2
Henan	7	2	6	2
Hubei	1	3	4	3
Hunan	2	0	0	0
Japan	0	0	2	0
Jiangsu	11	1	4	4
South Korea	0	0	8	1
Shanxi	1	0	2	1
Shandong	9	0	11	1
Sichuan	7	2	10	8
Taiwan	0	1	0	0
Thailand	0	0	1	1
USA	0	0	2	0
Zhejiang	1	1	2	1
Total	54	63	80	39

**Table 2:** Comparison between the model-based groups.

Group	Landraces	Modern cultivars	*P*_*i*_	Number of Core sets
Group 1	0	54	0.333	9
Group 2	50	13	0.338	14
Group 3	0	80	0.319	16

*P*_*i*_: Nucleotide diversity

**Fig 4:**
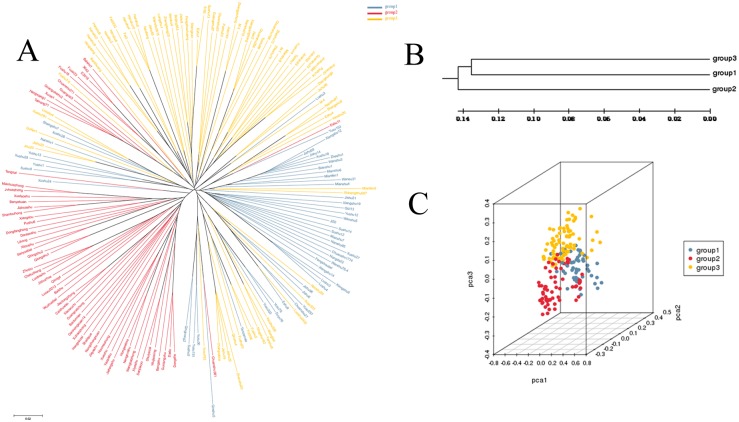
Characterization of the genetic structure of the 197 sweet potato accessions. (A) Phylogenetic tree of the 197 accessions based on the analysis of 62,363 SNPs. (B) UPGMA dendrogram based on Nei’s distance among three inferred groups. (C) PCA plot of the 197 accessions based on the analysis of 62,363 SNPs.

Neighbor-joining cluster analysis clearly divided the 197 accessions into three groups (Fig 4A); this result was consistent with the assignments made using ADMIXTURE. The UPGMA dendrogram of the 197 accessions revealed that group 1 was genetically more similar to group 3 than to group 2 (Fig 4B). Group 1 and group 3 clustered together with a genetic distance of 0.271, while group 2 stood alone, exhibiting a relatively large genetic distance from the other groups (i.e., 0.284 for group 1 and 0.288 for group 3) (Table 3). The PCA also separated the 197 accessions into three major groups (Fig 4C). Nucleotide diversity (*P*_*i*_) indicated that the accessions in group 2 exhibited a higher genetic diversity than those in group 1 and group 3 (Table 2). The three groups were intermixed.

**Table 3:** Genetic distance (Downward Diagonal) and pairwise F_*st*_ (Upward Diagonal) among the three groups inferred through structure analysis.

	Group 1	Group 2	Group 3
Group 1		0.069	0.045
Group 2	0.284		0.046
Group 3	0.271	0.288	

### Genetic diversity

Genetic parameters, including genetic distance, PIC and MAF, were estimated separately to evaluate the genetic diversity of the three groups (Table 4; S1 Fig). The highest mean genetic distance was present in group 3 (0.311), and the lowest was found in group 1 (0.290). Comparison of the mean PIC revealed that group 3 was highly polymorphic, whereas group 1 exhibited the lowest PIC. The mean MAF across the three groups ranged from 0.207 to 0.222.

**Table 4:** Diversity statistics for the 197 sweet potato accessions collected in this study.

Group	Genetic distance		PIC		MAF	
	Mean	Range	Mean	Range	Mean	Range
Group 1	0.290	0~0.524	0.232	0~0.375	0.207	0~0.500
Group 2	0.307	0~0.527	0.246	0~0.375	0.219	0~0.500
Group 3	0.311	0~0.512	0.251	0~0.375	0.222	0~0.500

PIC: Polymorphic information content

MAF: Minor allele frequency

### Population differentiation

A population differentiation analysis was performed to analyze the genetic variations among and within groups, as revealed by the population structure. AMOVA revealed that the maximum diversity of 89.569% occurred within accessions, while the minimum diversity of 3.152% was attributed to genetic differentiation among groups (Table 5). The pairwise F_*st*_ analysis among the three inferred groups indicated that group 1 and group 2 showed the highest differentiation, with an F_*st*_ of 0.069; group 1 and group 3 were the most closely related, with an F_*st*_ of 0.045 (Table 3), this corroborated the results of UPGMA.

**Table 5:** AMOVA of the 197 sweet potato accessions collected in this study.

Source of variation	Df	Sum of squares	Mean square	Sigma	Components of covariance (%)
Among groups	2	29,001.660	14,500.830	89.178	3.152
Among accessions within groups	194	571,656.800	2946.684	205.996	7.279
Within accessions	197	499,334.400	2534.692	2534.692	89.569
Total	393	1,099,992.860	2798.964	2829.866	100.00

### Development of a core set

**Fig 5:**
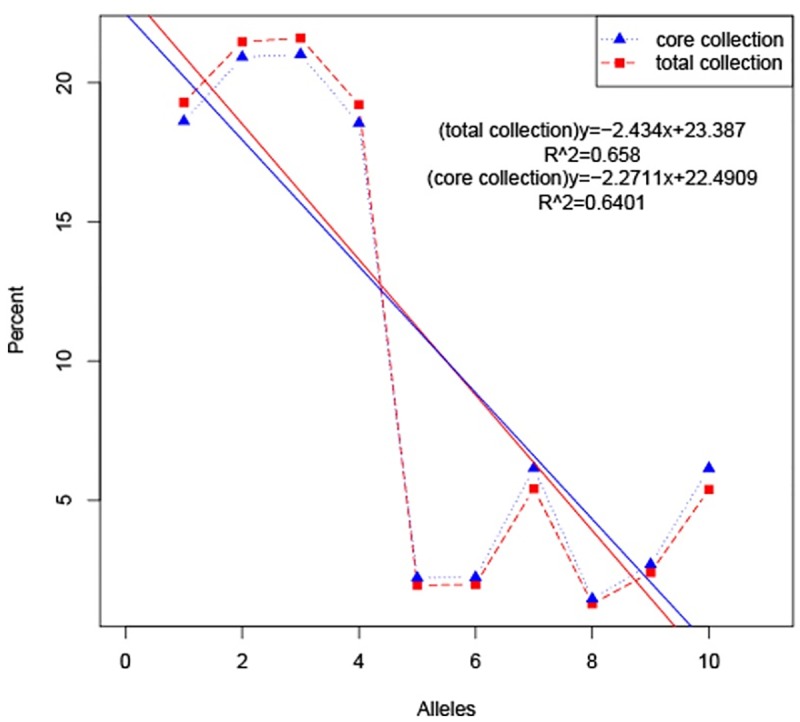
Comparison of the frequency of occurrence of alleles in the total collection *versus* the core set. The x-axis indicated ten allele types, (1) AA; (2) CC; (3) GG; (4) TT; (5) GT/TG; (6) AC/CA; (7) AG/GA; (8) CG/GC; (9) AT/TA; (10) CT/TC. The y-axis showed the allele frequencies.

Among the 197 sweet potato accessions studied, a core set of 39 accessions was selected using CoreHunter software (S3 Table), three of which were from Africa, South Korea and Thailand, while the remainder were from different provinces of China. Among these core set accessions, nine accessions were from group 1, 14 from group 2, 16 from group 3 (Tables 1 and 2). Ten allele types were produced, of which 4 alleles were homozygous and 6 were heterozygous. Allele frequencies were calculated for the total collection and the core set, and the results showed that there were no loss of alleles in the resulting core set (Table 6; Fig 5). Genetic diversity parameters and population structure were analyzed for the core set.

**Table 6:** Comparison of percent of alleles generated in the total collection v*ersus* the core set.

	Alleles									
	AA	CC	GG	TT	GT/TG	AC/CA	AG/GA	CG/GC	AT/TA	CT/TC
Total collection (197 acc.)	19.29	21.47	21.60	19.21	1.95	1.97	5.42	1.28	2.40	5.39
Core set (39 acc.)	18.61	20.92	21.02	18.54	2.22	2.23	6.15	1.46	2.70	6.15

### Genetic diversity of the core set

**Fig 6:**
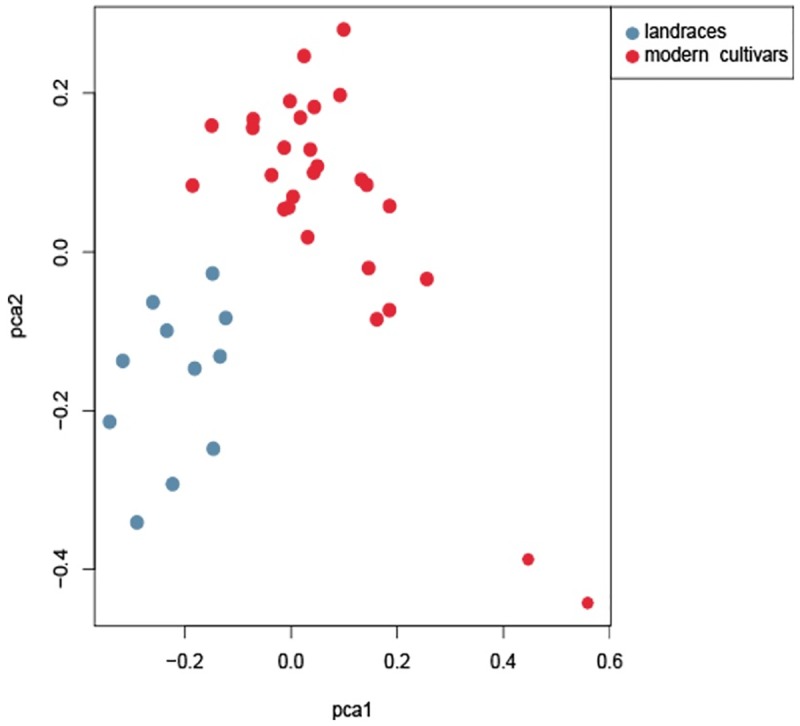
PCA of the sweet potato core set.

The genetic diversity of the core set was estimated to determine the extent of diversity captured from the total collection. Comparisons of all genetic parameters revealed that the values for the core set were greater than those for the total collection (Table 7). For example, the mean genetic distance of the total collection was 0.303, but this value increased to 0.319 in the core set. Similarly, the mean PIC and the mean MAF of the total collection were 0.243 and 0.216, while those of the core set were 0.255 and 0.226, respectively. PCA clustering showed that the accessions of the core set were distributed into two clusters, with the landraces distanced from the modern cultivars (Fig 6).

**Table 7:** Comparison of the genetic diversity of the total collection *versus* the core set.

Genetic distance				PIC				MAF			
Total collection (197 acc.)		Core set (39 acc.)		Total collection (197 acc.)		Core set (39 acc.)		Total collection (197 acc.)		Core set (39 acc.)	
Mean	Range	Mean	Range	Mean	Range	Mean	Range	Mean	Range	Mean	Range
0.303	0~0.527	0.319	0~0.520	0.243	0~0.375	0.255	0~0.375	0.216	0~0.500	0.226	0~0.500

PIC: Polymorphic information content

MAF: Minor allele frequency

### AMOVA of the core set

AMOVA of the core set showed 3.876% of the variance among groups (Table 8); in contrast, 11.954% of the variance was present among accessions within groups, and the maximum diversity occurred within accessions. The partitioning of molecular variance was similar to that in the total collection (Table 5). A plot of the percentage allele frequency for the core set *versus* the total collection showed that nearly all alleles in the total collection were represented in the core set with similar frequencies (Fig 5).

**Table 8:** AMOVA of the sweet potato core set.

Source of variation	Df	Sum of squares	Mean square	Sigma	Components of covariance (%)
Among groups	3	30,834.410	10,278.136	221.948	3.876
Among accessions within groups	35	216,587.900	6188.226	684.459	11.954
Within accessions	39	187,953.000	4819.308	4819.308	84.170
Total	77	435,375.310	5654.225	5725.715	100.00

## Discussion

### SNP-based assessment of population structure

The genetic architecture of diverse sweet potato accessions was precisely estimated using 62,363 SNPs. In this study, the concordance of the model-based structure analysis revealing three groups in the population (Fig 2) with the phylogenetic tree and PCA clustering agreed with the results of Yang et al. [17], which were generated using SSRs. Furthermore, for landraces and modern cultivars, the assignment to groups was basically in accordance with the previous study using SSRs, which also clustered landraces separately from modern cultivars (Table 2) [17]. This indicates, not surprisingly, that the landraces are genetically distant from modern cultivars. The calculation of genetic distance between groups shown in Table 3 confirms this result. Nevertheless, the clustering of the 197 accessions according to the phylogenetic tree or PCA and their assignment into groups did not agree with the information on their geographic origin. This discrepancy can be explained by the acceleration of germplasm resource exchange between regions; many previous studies also support these results [7,11,12,14].

The UPGMA dendrogram based on Nei’s distance among the three inferred groups (Fig 4B) indicated that group 2, which was mostly composed of landraces, was genetically distant from group 1 and group 3. This distance may be due to the breeding history of sweet potato; it is possible that the landraces of group 2 originated overseas and dispersed across a broad region of China, resulting in group 1 and group 3. This pattern also agrees with the results obtained by Yang et al. [17]. Thus, we inferred that the sweet potato population genetic structures estimated based on SNPs and SSRs were similar.

Model-based AMOVA showed that the maximum diversity of 89.569% occurred within accessions, while the minimum diversity of 3.152% was attributed to genetic differentiation among groups (Table 5). Yang et al. [17] performed AMOVA on 380 sweet potato accessions and revealed 16.47% variation among groups and 83.53% variation within accessions. The variation within accessions observed in our study was higher than that reported previously, possibly due to the large number of markers developed in the present study.

### Genetic diversity assessed based on SNPs

Long-term selection gain requires genetic variability; thus, it is important to examine not only population structure but also genetic diversity [40]. Across the 197 sweet potato accessions examined in this study, we observed mean genetic distances of 0.290 in group 1, 0.307 in group 2, and 0.311 in group 3; mean PICs of 0.232 in group 1, 0.246 in group 2, and 0.251 in group 3; and mean MAFs of 0.207 in group 1, 0.219 in group 2, and 0.222 in group 3 (Table 4). Yang et al. [17] reported that the average genetic distances of 380 sweet potato accessions (as determined by SSRs) ranged from 0.220 to 0.254, while the average PIC ranged from 0.181 to 0.204. The higher genetic diversity observed in this study might be explained by the number of SNPs evaluated and the combination of SNP alleles at different loci.

### Development of the core germplasm set

Recently, various types of molecular markers have been used to develop core germplasm sets for different types of crops. These markers include RAPD for common bean [41] and Spanish melon [42]; AFLPs for barley [43]; SSRs for rice, wheat, common bean and olive [44–47]; and SNPs for olive and *Arabidopsis* [47,48]. In this study, genomic characterization revealed high genetic diversity within the 197 sweet potato accessions; therefore, we decided to identify a core germplasm set to aid sweet potato breeders in effectively using the accessions in their crop improvement programs. This was the first time that SNPs have been used to identify major variances and select a fully representative germplasm set from a large sweet potato collection.

CoreHunter software was employed in this study to develop a core germplasm set of 39 sweet potato accessions, accounting for 19.8% of the total collection. However, the sampling percentage of the core germplasm set to fit all crops has long been under debate, possibly due to the large extent of germplasm resources and the complex types of data involved [9]. A sampling percentage of 20~30% was once suggested by Yonezawa et al. [49], and mini core sets representing approximately 1% of total collections have been used to characterize very large collections [50,51]. A perfect ratio and fixed size for all core germplasm sets do not exist because different crops and different goals require different sampling percentages [9]. In the current study, nearly all alleles were represented in the core set (Fig 5); thus, this was the perfect sampling percentage for this study.

Genetic parameters and cluster analysis were used to evaluate the efficiency of the development of the core germplasm set; these methods have been described in many other reports [9,52–55]. In the present study, the mean genetic distance, PIC, and MAF values of the core germplasm set were higher than those of the total collection (Table 7), which was expected because diversity increases after the elimination of genetically similar accessions during core germplasm set development [56]. Cluster analysis clearly separated the core set into two groups according to their types; these groups were distributed separately into a two-dimensional plot for PCA (Fig 6). AMOVA based on this model showed that the partitioning of molecular variance was similar to that for the total collection. The germplasm core set developed in this study was statistically supported by the above genetic analyses.

## Conclusion

To the best of our knowledge, the SNPs reported in this study are the most saturated markers yet obtained for sweet potato. The SNP-based molecular characterization of the sweet potato collection in this study revealed large variations within accessions. The pattern of population structure and genetic diversity varied across model-based groups. Group 2, which consisted mostly of landraces, was genetically distant from the other two groups. A core germplasm set of 39 sweet potato accessions, accounting for 19.8% of the total collection, was developed. The genome-level profiling of 197 sweet potato accessions and the development of the core set in this study will provide a foundation for genomic studies and for the identification of potential parents for sweet potato improvement.

## Supporting information

S1 Fig Boxplots of genetic diversity among the three groups. (TIF)

S1 Table Regional distribution of the 197 sweet potato accessions collected in this study. (DOC)

S2 Table List of SNPs developed in this study. (SNPLIST)

S3 Table List of sweet potato accessions identified for the core collection based on SNPs. (XLS)
