# Supplementary material for: Genome-wide assessment of population structure and genetic diversity and development of a core germplasm set for sweet potato based on specific length amplified fragment (SLAF) sequencing
Source: PLoS One. 2017 Feb 10;12(2):e0172066. doi: 10.1371/journal.pone.0172066 (PMC5302839; doi:10.1371/journal.pone.0172066)
Supplement: S1 Table — (DOC) [file pone.0172066.s002.doc]

**S1 Table Regional Distribution of the 197 Sweet Potato Accessions Collected in This Study**

| No: | Sample Code | Variety | Origin | Group | Type |
| --- | --- | --- | --- | --- | --- |
| 1 | eq | FeiSOD | Africa | 3 | MC |
| 2 | iq | M-4 | Africa | 3 | MC |
| 3 | iv | Fei3 | Africa | 3 | MC |
| 4 | ju | M-1 | Africa | 3 | MC |
| 5 | jx | Tanzannia | Africa | 1 | MC |
| 6 | kh | M-3 | Africa | 3 | MC |
| 7 | cr | Wanshu3 | Anhui | 1 | MC |
| 8 | cu | Wanshu5 | Anhui | 1 | MC |
| 9 | ke | Wanshu78-4 | Anhui | 1 | MC |
| 10 | fq | Nongda22 | Beijjing | 1 | MC |
| 11 | iu | Yizi306 | Beijjing | 3 | MC |
| 12 | ix | Beijing553 | Beijjing | 3 | MC |
| 13 | kg | Wansu31 | Chongqing | 1 | MC |
| 14 | az | Yusu297 | Chongqing | 1 | MC |
| 15 | bf | Yusu76 | Chongqing | 1 | MC |
| 16 | bk | Yusu30 | Chongqing | 1 | MC |
| 17 | bm | Yusu153 | Chongqing | 1 | MC |
| 18 | bq | Yushu99 | Chongqing | 3 | MC |
| 19 | br | Yusu162 | Chongqing | 3 | MC |
| 20 | dm | Suyu303 | Chongqing | 3 | MC |
| 21 | eg | Yusu151 | Chongqing | 1 | MC |
| 22 | hs | Wanshu0515 | Chongqing | 3 | MC |
| 23 | ij | Yusu303 | Chongqing | 1 | MC |
| 24 | af | Pushu53 | Fujian | 3 | MC |
| 25 | ah | Fushu18 | Fujian | 2 | MC |
| 26 | aj | Fushu7-6 | Fujian | 3 | MC |
| 27 | bv | Fushu14 | Fujian | 3 | MC |
| 28 | dc | Pushu14 | Fujian | 3 | MC |
| 29 | fz | Pushu6 | Fujian | 2 | L |
| 30 | gw | Longshu1 | Fujian | 3 | MC |
| 31 | is | Fushu2 | Fujian | 3 | MC |
| 32 | iy | Fushu20 | Fujian | 3 | MC |
| 33 | km | Fucai23 | Fujian | 2 | MC |
| 34 | kq | Quanshu23 | Fujian | 3 | MC |
| 35 | ac | Guangcai3 | Guangdong | 2 | MC |
| 36 | db | Guangshu87 | Guangdong | 3 | MC |
| 37 | dz | Guifen1 | Guangdong | 3 | MC |
| 38 | fs | Baidumian | Guangdong | 2 | L |
| 39 | ft | Sanyuebai | Guangdong | 2 | L |
| 40 | fu | Luoboshu | Guangdong | 2 | L |
| 41 | fw | Cheluzhong | Guangdong | 2 | L |
| 42 | fx | Jinshan25 | Guangdong | 3 | MC |
| 43 | fy | Jizhuzhua | Guangdong | 2 | L |
| 44 | ga | Nengnvshu | Guangdong | 2 | L |
| 45 | gd | Dongshu | Guangdong | 2 | L |
| 46 | ge | Hongxinzai | Guangdong | 2 | L |
| 47 | gg | Xiangshu | Guangdong | 2 | L |
| 48 | gh | Xiyeshu | Guangdong | 2 | L |
| 49 | gi | Dongfanghong | Guangdong | 2 | L |
| 50 | gj | Yazaishu | Guangdong | 2 | L |
| 51 | gk | Wuzhuabai | Guangdong | 2 | L |
| 52 | gl | Bangshu | Guangdong | 2 | L |
| 53 | gn | Xuhaozhong | Guangdong | 2 | L |
| 54 | go | Niangzaizhong | Guangdong | 2 | L |
| 55 | gp | Guangcaizhong | Guangdong | 2 | L |
| 56 | gq | Hongweima | Guangdong | 2 | L |
| 57 | gr | Zhutou | Guangdong | 2 | L |
| 58 | gs | Danlanshu | Guangdong | 2 | L |
| 59 | gt | Shalayue | Guangdong | 2 | L |
| 60 | gu | Jiayashu | Guangdong | 2 | L |
| 61 | gz | Shantouhong | Guangdong | 2 | L |
| 62 | ha | Xitoushu | Guangdong | 2 | L |
| 63 | hb | Guniangshu | Guangdong | 2 | L |
| 64 | he | Caishuxian | Guangdong | 2 | L |
| 65 | hf | Xiaowuchi | Guangdong | 2 | L |
| 66 | hg | Jiefangshu | Guangdong | 2 | L |
| 67 | hi | Banyekuan | Guangdong | 2 | L |
| 68 | hj | Gaonongxuan14 | Guangdong | 2 | L |
| 69 | kp | Jvhuazhong | Guangdong | 2 | L |
| 70 | kr | Xueshu | Guangdong | 2 | L |
| 71 | ks | Dadu | Guangdong | 2 | L |
| 72 | kv | Houmaohong | Guangdong | 2 | L |
| 73 | kw | Jiaotongzhong | Guangdong | 2 | L |
| 74 | kx | Guangcaishu2 | Guangdong | 2 | MC |
| 75 | ky | Shuiyezai | Guangdong | 2 | L |
| 76 | kz | Xuelaoshu | Guangdong | 2 | L |
| 77 | la | Lihong | Guangdong | 2 | L |
| 78 | lb | Jinshan57 | Guangdong | 3 | MC |
| 79 | lc | Sanjiaoyu | Guangdong | 2 | L |
| 80 | le | Jizhuashu | Guangdong | 2 | L |
| 81 | lf | Wujiaoxing | Guangdong | 2 | L |
| 82 | lg | Baishu | Guangdong | 2 | L |
| 83 | lh | Nongzhongxuan | Guangdong | 2 | L |
| 84 | li | Tengzai | Guangdong | 2 | L |
| 85 | lj | Jinshan1885 | Guangdong | 3 | MC |
| 86 | ll | Mianhuazhong | Guangdong | 2 | L |
| 87 | hq | Qiongshu1 | Hainan | 2 | L |
| 88 | hu | Qiongshu2 | Hainan | 2 | L |
| 89 | jb | Qiong4 | Hainan | 2 | L |
| 90 | da | Jishu332 | Hebei | 3 | MC |
| 91 | do | Jishu65 | Hebei | 3 | MC |
| 92 | dr | Jishu46 | Hebei | 3 | MC |
| 93 | dv | Jishu98 | Hebei | 1 | MC |
| 94 | ec | Jishu6 | Hebei | 1 | MC |
| 95 | fb | Weiduoli | Hebei | 3 | MC |
| 96 | hr | Jishu99 | Hebei | 1 | MC |
| 97 | be | Xiangshu14 | Henan | 1 | MC |
| 98 | bg | Xiangshu6 | Henan | 1 | MC |
| 99 | by | Yushu12 | Henan | 1 | MC |
| 100 | cz | Baishu1 | Henan | 2 | MC |
| 101 | dd | Zhenghong9 | Henan | 3 | MC |
| 102 | dg | Zhenshu20 | Henan | 3 | MC |
| 103 | dh | Luoshu0402 | Henan | 3 | MC |
| 104 | dn | Yushu13 | Henan | 1 | MC |
| 105 | eh | Shangshu7 | Henan | 1 | MC |
| 106 | el | Zhengyinhong | Henan | 3 | MC |
| 107 | ev | Zhenghong20 | Henan | 3 | MC |
| 108 | fm | Bianshu1 | Henan | 1 | MC |
| 109 | hv | Yushu1 | Henan | 1 | MC |
| 110 | iw | Luoxu0213 | Henan | 2 | L |
| 111 | kk | Shangshu9 | Henan | 3 | MC |
| 112 | ap | Eshu11 | Hubei | 2 | MC |
| 113 | jc | Eshu9 | Hubei | 1 | MC |
| 114 | jd | E3018 | Hubei | 2 | MC |
| 115 | jk | Eshu1 | Hubei | 3 | MC |
| 116 | jl | Eshu4 | Hubei | 3 | MC |
| 117 | jm | Luoshu4 | Hubei | 3 | MC |
| 118 | js | 3043 | Hubei | 2 | MC |
| 119 | jt | 5193 | Hubei | 3 | MC |
| 120 | ax | Xiangshu19 | Hunan | 1 | MC |
| 121 | ay | Xiangshu12 | Hunan | 1 | MC |
| 122 | bu | Hongdong | Japan | 3 | MC |
| 123 | fc | Nanfeng | Japan | 3 | MC |
| 124 | ab | Xushu781 | Jiangsu | 3 | MC |
| 125 | ad | Sushu9 | Jiangsu | 1 | MC |
| 126 | ak | Xucai1 | Jiangsu | 2 | MC |
| 127 | ba | Nanshu1 | Jiangsu | 1 | MC |
| 128 | bb | Sushu13 | Jiangsu | 3 | MC |
| 129 | ce | Sushu14 | Jiangsu | 1 | MC |
| 130 | ch | Sushu5 | Jiangsu | 1 | MC |
| 131 | cs | Suyu76 | Jiangsu | 1 | MC |
| 132 | de | Xushu28 | Jiangsu | 1 | MC |
| 133 | dj | Ningshu192 | Jiangsu | 3 | MC |
| 134 | dp | Xushu27 | Jiangsu | 1 | MC |
| 135 | ew | Mengbai | Jiangsu | 3 | MC |
| 136 | hk | Sushu12 | Jiangsu | 1 | MC |
| 137 | hw | Xushu26 | Jiangsu | 1 | MC |
| 138 | hx | Xushu18 | Jiangsu | 1 | MC |
| 139 | ko | Xushu24 | Jiangsu | 1 | MC |
| 140 | em | Hanshu3 | South Korea | 3 | MC |
| 141 | en | Hanshu2 | South Korea | 3 | MC |
| 142 | eo | Hanshu4 | South Korea | 3 | MC |
| 143 | ep | Hanshu6 | South Korea | 3 | MC |
| 144 | hp | Hanshu8 | South Korea | 3 | MC |
| 145 | jo | Hanshu10 | South Korea | 3 | MC |
| 146 | jp | Hanshu5 | South Korea | 3 | MC |
| 147 | kj | Hanshu9 | South Korea | 3 | MC |
| 148 | du | Qinshu4 | Shanxi | 3 | MC |
| 149 | ed | Jishu21 | Shandong | 1 | MC |
| 150 | ef | Qizi13 | Shandong | 1 | MC |
| 151 | ek | Yanshu20 | Shandong | 3 | MC |
| 152 | er | Ji20 | Shandong | 1 | MC |
| 153 | et | Qingnong2 | Shandong | 1 | MC |
| 154 | eu | Jishu22 | Shandong | 3 | MC |
| 155 | ex | Lushu3 | Shandong | 1 | MC |
| 156 | fa | Yanshu8 | Shandong | 3 | MC |
| 157 | fh | Jishu19 | Shandong | 3 | MC |
| 158 | fr | Jishu8 | Shandong | 3 | MC |
| 159 | ht | Jishu14 | Shandong | 1 | MC |
| 160 | ie | Banlihuang | Shandong | 3 | MC |
| 161 | if | Jixu23 | Shandong | 3 | MC |
| 162 | ig | Lushu2 | Shandong | 1 | MC |
| 163 | ii | Fengshouhuang | Shandong | 3 | MC |
| 164 | it | Lushu4 | Shandong | 1 | MC |
| 165 | iz | Yanshu526 | Shandong | 3 | MC |
| 166 | je | Changcaixia | Shandong | 3 | MC |
| 167 | kf | Jishu23 | Shandong | 3 | MC |
| 168 | kn | Fengshoubai | Shandong | 1 | MC |
| 169 | df | Xiangyanghong | Shanxi | 3 | MC |
| 170 | dy | Qinshu5 | Shanxi | 1 | MC |
| 171 | am | Chuanshu211 | Sichuan | 2 | MC |
| 172 | av | Chuanshu20 | Sichuan | 3 | MC |
| 173 | bc | Chuanshu294 | Sichuan | 3 | MC |
| 174 | bd | Chuanshu1774 | Sichuan | 1 | MC |
| 175 | bo | Chuanshu168 | Sichuan | 3 | MC |
| 176 | cc | Chuanshu27 | Sichuan | 1 | MC |
| 177 | cd | Mianshu8 | Sichuan | 3 | MC |
| 178 | cf | Nanshu95 | Sichuan | 1 | MC |
| 179 | ci | Mianshu7 | Sichuan | 1 | MC |
| 180 | cl | Shengnan | Sichuan | 3 | MC |
| 181 | cn | Mianshu4 | Sichuan | 1 | MC |
| 182 | cp | Nanshu010 | Sichuan | 3 | MC |
| 183 | cq | Chuanshu381 | Sichuan | 2 | MC |
| 184 | cv | Mianfen1 | Sichuan | 1 | MC |
| 185 | cw | Mianfen5 | Sichuan | 3 | MC |
| 186 | cx | Mianshu6 | Sichuan | 1 | MC |
| 187 | dt | Nanshu99 | Sichuan | 3 | MC |
| 188 | hm | Nanshu97 | Sichuan | 3 | MC |
| 189 | im | Xichengshu007 | Sichuan | 3 | MC |
| 190 | ag | Tainong71 | Taiwan | 2 | MC |
| 191 | jq | Taishu1 | Thailand | 3 | MC |
| 192 | dw | Jiao140 | USA | 3 | MC |
| 193 | kd | Jiao2 | USA | 3 | MC |
| 194 | ai | Hangxiang1 | Zhejiang | 2 | MC |
| 195 | cj | Zheshu13 | Zhejiang | 3 | MC |
| 196 | dk | Zheshu70 | Zhejiang | 3 | MC |
| 197 | ki | Zheshu1 | Zhejiang | 1 | MC |

L = landrace;

MC = modern cultivar.
